# Power, Politics, and Policy in the Making of Health Inequities: An Empirical Study of Equity-Oriented Policy Actors in Canada

**DOI:** 10.1177/27551938261467630

**Published:** 2026-07-10

**Authors:** Arnel M. Borras

**Affiliations:** 1 Rankin School of Nursing, 1270 St. Francis Xavier University, 4130 University Avenue, Antigonish, Nova Scotia B2G 2W5, Canada

**Keywords:** health equity policy, critical political economy of health, structural power, corporate power and influence, neoliberal governance, democratic constraints, redistributive reform, civil society, policymaking, Canada

## Abstract

Structural health inequities in Canada remain entrenched—and in some areas have intensified—despite decades of commitments to equity. This study examines how power relations shape the conditions under which health equity policy reform is pursued and constrained. Grounded in critical political economy, the analysis draws on 23 semi-structured interviews with Canadian policy scholars, advocates, union leaders, and health professionals engaged in equity-oriented work. Thematic analysis identified four interrelated dynamics shaping health equity policymaking: institutional arrangements that privilege capital accumulation over redistribution; the institutionalization of corporate power within policymaking; neoliberal governance norms that narrow politically feasible policy responses; and fragmentation within civil society that weakens sustained collective pressure for redistributive change. Together, these dynamics illuminate how power is organized across economic, political, and institutional domains, contributing to persistent health inequities despite formal equity commitments. While participants referenced participation, consultation, and access to decision making, these mechanisms were experienced within policy environments characterized by structural power asymmetries. Rather than attributing inequities to evidence gaps or implementation failures, the analysis situates them as outcomes of political–economic arrangements shaping policy feasibility. Advancing health equity therefore requires confronting structural power relations and strengthening collective capacity for redistributive and democratic change beyond the health sector.

Despite decades of policy attention and formal commitments to equity, health inequities in Canada remain deeply entrenched across social groups. A substantial body of classical and contemporary research demonstrates that these inequities are structurally produced through intersecting hierarchies of class, race, gender, and other social relations.^1-5^ While there is broad consensus on the importance of addressing the social determinants of health (SDH)—including employment, income, housing, and access to health services^6,7^—dominant policy frameworks often sideline the political and institutional forces that shape state action, resource distribution, and ultimately, health outcomes.^8,9^ The persistence of health inequities therefore calls for a closer examination of the power relations that structure policymaking, govern resource distribution, and constrain equity-oriented reform.

This study is grounded in a critical political economy analysis of equity policymaking in Canada. From this perspective, health policy making is understood as a structurally uneven terrain shaped by capital accumulation, institutionalized power asymmetries, and historically entrenched governance arrangements.^1,6-9^ Pluralism is not adopted as an explanatory framework in this analysis. Instead, pluralist assumptions are engaged reflexively as empirical points of contrast, examining how ideals of openness, access, and democratic responsiveness are experienced and interpreted by equity policy actors operating within contemporary institutional contexts.

Classical pluralism conceptualizes equity policymaking as an open and competitive arena in which diverse interests exert influence over policy outcomes through negotiation and compromise.^10-12^ Long-standing critiques of pluralism, however, document the disproportionate influence of elite actors—particularly corporate interests—over policy agendas, institutional norms, and redistributive possibilities, raising fundamental questions about democratic participation and political equality.^13-17^ These dynamics are reinforced by scholarship on the commercial determinants of health, which demonstrates how corporate actors shape regulation, research agendas, and public discourse in ways that prioritize profit over population health.^18-19^ In this study, these pluralist assumptions are examined empirically through participants’ accounts of power, constraint, and exclusion within health and social policymaking processes.

By foregrounding power relations, this article contributes to politically engaged public health scholarship that seeks to move beyond descriptive accounts of inequity toward explanations rooted in political economy.^1,6-9,20-24^ It centers the perspectives of Canadian policy scholars, advocates, and activists navigating structurally unequal policy terrains, generating empirical insight into how equity is conceptualized, pursued, and constrained in practice. Guided by the research question “How do policy actors interpret and navigate equity policymaking within contemporary political and institutional constraints?” the study examines how participants engage governance structures, corporate influence, advocacy coalitions, and institutional norms—illuminating the conditions that shape the limits of advancing health equity policy within prevailing political–economic arrangements.

This article is organized as follows. The Methods section contains the study design, participant recruitment, data collection, and analytic approach. The Findings section comprises four empirical themes derived from participants’ accounts. The Discussion section contains interpretations of these themes through a critical political economy lens attentive to capital accumulation, institutional power, and democratic constraint. The final section contains an examination of the implications for health equity policymaking and concludes the article.

## Methods

This study employed a qualitative interview-based design to examine how equity-oriented policy actors interpret and navigate health equity policymaking within contemporary political and institutional settings in Canada. Semi-structured interviews were selected to generate in-depth accounts of participants’ experiences, interpretations, and strategic reflections, allowing for exploration of power, governance, and structural constraint as lived and practical phenomena.

### Study Design and Participants

This study draws on 23 semi-structured interviews with Canadian policy scholars, advocates, and activists and labor leaders engaged in public policy, health equity, and social determinants of health work. Participants were recruited using purposive sampling to identify individuals with recognized expertise in health inequities, public policy, political economy, and equity-oriented advocacy (*n* = 20). Three additional participants (*n* = 3) were recruited through chain-referral sampling. Of 42 individuals invited to participate, 23 agreed, yielding a response rate of 55%. Recruitment aimed to capture diversity across sector, discipline, and professional role.

Participants were drawn from academia, public health, labor unions, community organizations, and advocacy groups. Of the participants, 9 had primary expertise in health-related fields, 10 in broader public policy and political economy, and 4 were union leaders within the health sector. Professional backgrounds spanned epidemiology, sociology, political science, political economy, economics, medicine, nursing, housing policy, labor studies, and community organizing.

The sample comprised 15 men and 8 women. Eighteen participants were visibly white, and 5 were visibly people of color. One participant was publicly identified as Métis, and one self-identified as Jewish. These demographic characteristics reflect response pattern rather than sampling criteria and are reported to enhance transparency regarding sample composition. Five participants requested anonymity. Table 1 contains a summary of participants’ sectoral affiliations, areas of expertise, and roles.

**Table 1.** Study Participants by Sector, Expertise, and Role.

Participant Name	Sector/Role	Health Focus	Non-Health Focus	Labor Affiliation	Primary Expertise	Affiliation / Position
John Clarke	Activist	No	Yes	No	Poverty, Housing, Homelessness, Activism	Former Organizer, OCAP
William Carroll	Academic	No	Yes	No	Sociology, Political Ecology, Social Movements	Professor, UVic
Trevor Hancock	Academic, Advocate	Yes	No	No	Public Health, SDH, Health Promotion	Professor Emeritus, UVic, Green Party Founding Leader
Cathy Crowe	Activist	No	Yes	No	Housing, Homelessness, Activism	Founder, Shelter & Justice Network
Anon N5	Academic, Advocate	Yes	No	No	Economics, Labor, Health	—
David Hulchanski	Academic	No	Yes	No	Housing, Urban Planning Social Inequality	Professor, U of T
Anon N7	Academic	No	Yes	No	Sociology, Gender, Race, Migration	—
Leo Panitch^†^	Academic	No	Yes	No	Political Science, Socialism	Professor Emeritus, YorkU
Pat Armstrong	Academic, Advocate, Activist	Yes	No	No	Sociology, Healthcare, Gender	Professor Emeritus, YorkU
Sam Gindin	Activist	No	Yes	No	Political Economy, Labor	Former Research Director, CAW
Anon N11	Advocate, Activist	No	No	Yes	Labor and Healthcare	Trade Union Leader
Luin Goldring	Academic	No	Yes	No	Labor, Migration, Precarity, Citizenship	Professor, YorkU
Kwame McKenzie	Academic, Advocate, Activist	Yes	No	No	Health Equity, SDH, Policy	CEO, Wellesley Institute; Director of Health Equity, CAMH; Professor, U of T
Anon N14	Academic, Advocate	Yes	No	No	Epidemiology, Health Equity, SDH	—
Sheila Block	Advocate	No	Yes	No	Public Finance, Labor, Policy Advocacy	Senior Economist, CCPA
Akwatu Khenti	Academic, Advocate, Activist	Yes	No	No	Health Equity, SDH, Policy	Associate Professor, U of T; CAMH Centre for Global Health
Sharleen Stewart	Activist, Advocate	No	No	Yes	Labor and Healthcare	Former President, SEIU Healthcare Local 1
Greg Albo	Academic, Advocate, Activist	No	Yes	No	Political Science, Political Economy	Associate Professor, YorkU
Anon N19	Academic, Activist	Yes	No	No	Health Equity, SDH	—
Natalie Mehra	Advocate	Yes	No	No	Healthcare, Policy, Advocacy	Executive Director, Ontario Health Coalition
Michael Hurley	Activist	No	No	Yes	Labor and Healthcare	President, OCHU; Regional VP, CUPE
Jonah Gindin	Advocate, Activist	No	No	Yes	Collective Bargaining, Labor, Healthcare	Researcher, CUPE Ontario OCHU
Toba Bryant	Academic	Yes	No	No	Health Equity, SDH, Policy	Associate Professor, Ontario Tech University
		9	10	4		

**Notes**: *Anon N#* indicates participant chose anonymity. Abbreviations: OCAP = Ontario Coalition Against Poverty; UVic = University of Victoria; YorkU = York University; U of T = University of Toronto; CAMH = Centre for Addiction and Mental Health; CCPA = Canadian Centre for Policy Alternatives; CAW = Canadian Auto Workers; SEIU = Service Employees International Union; OCHU = Ontario Council of Hospital Unions; CUPE = Canadian Union of Public Employees; † Deceased.

### Data Collection

Interviews were conducted between January and March 2020. Eight interviews were conducted in person, 6 by telephone, and 9 online, with modality shaped in part by emerging public health restrictions related to the COVID-19 pandemic. Interviews ranged from approximately 30 to 120 min, with most lasting between 45 and 60 min.

The interview guide explored participants’ perspectives on the structural drivers of health and social inequities, the role of state institutions in addressing the SDH, and the influence of power, politics, and corporate actors in policymaking processes. Participants were also invited to reflect on their own roles, strategies, and constraints within policy, academic, labor, and advocacy spaces. Interviews were conversational in tone, allowing participants to introduce issues they viewed as salient to equity policymaking.

All interviews were audio-recorded with informed consent and transcribed verbatim. Participants were offered the opportunity to review their transcripts prior to analysis. Ethics approval was obtained from York University's Research Ethics Board (Certificate STU 2019-135).

### Analytic Approach

Interview transcripts were analyzed using Braun and Clarke's six-phase approach to thematic analysis.^25^ This stand-alone method was selected for its flexibility and rigor in equity-oriented health research as well as its suitability for generating inductive themes while supporting theoretical interpretation.^26-29^ Initial coding was inductive and data-driven, focusing on participants’ language and framing, and recurring patterns across accounts. Coding was conducted using MAXQDA qualitative analysis software.

Following initial theme development, analytic interpretation was guided by critical political economy to examine how participants described capital accumulation, institutional power, corporate influence, governance norms, and collective action within policymaking processes. Pluralist assumptions about participation, access, and influence were engaged reflexively—as features of prevailing policy discourse—rather than treated as explanatory frameworks.

Initial thematic analysis of the full interview corpus was completed in December 2022. The analysis was subsequently revisited and refined for the present article, which was prepared for submission in April 2024. This iterative process ensured analytic coherence across sections and alignment between empirical findings and the study's theoretical orientation.

## Findings

Participants consistently emphasized structural conditions as shaping the scope and limits of equity-oriented reform. Across accounts, health inequities were linked by participants to broader political–economic arrangements influencing policy development and implementation. This section presents four empirical themes derived from participants’ accounts, outlining how interviewees interpreted and navigated the political and institutional forces shaping health equity policymaking in Canada. The themes are descriptive and grounded in participants’ professional and lived engagements with policy processes, illuminating how power operates within political–institutional contexts.

Despite variation in roles, sectors, and vantage points, participants articulated closely aligned interpretations of the conditions associated with social and health inequities. Thematic analysis identified four interrelated dynamics that participants repeatedly associated with the persistence of inequities and the constraints on transformative reform: (*a*) capital accumulation and enduring power asymmetries; (*b*) corporate dominance and the marginalization of equity agendas; (*c*) neoliberal state norms and the narrowing of democratic space; and (*d*) civil society fragmentation and the weakening of collective influence. These themes reflect the participants’ shared understanding of the political terrain within which equity agendas are pursued and constrained.

### Theme 1: Capital Accumulation and Enduring Power Asymmetries

Participants overwhelmingly located the roots of social and health inequities in capitalist political–economic structures and unequal distributions of power. Eighteen participants explicitly pointed to capital accumulation, concentration, and influence as structuring unequal access to health-promoting resources, while twelve emphasized unequal power relations as a core mechanism through which inequities are produced and sustained. Even participants who did not explicitly invoke “capital” or “capitalism,” nevertheless described market logics and corporate interests as shaping policy environments in ways that reproduce inequities.

Capitalism was commonly depicted not merely as an economic system, but as an institutional order organized around exploitation and extraction—one that concentrates wealth and decision-making authority while externalizing social and health harms. Poverty and ill health were framed as outcomes emerging from systemic conditions. John Clarke, activist and former OCAP organizer, observed:

“We live in a capitalist society . . . . The nature of that society . . . is fundamentally exploitative. There's a very small group of people who make a great deal of wealth, and there's a mass of people who essentially produce the wealth for them . . . . The whole strategy . . . is to ensure . . . those in the business of hiring workers . . . get this on the most favorable terms . . . . And so, there's more poverty and resultant assault on people's health.”

Several participants connected these dynamics to long-term patterns of privilege and dispossession, describing capital accumulation as expansionary and self-reinforcing. Unequal access to wealth, the means of production, and investment capacity was repeatedly identified as a mechanism through which inequities widen over time. As Anonymous N19, a physician, explained:

“Capitalism . . . is a major reason why we see things like poverty. Another intersecting aspect is the distribution of power and how that relates to capitalism . . . . Those who have wealth and the means of production . . . are able to continually invest that and get returns, and those who don’t . . . fall further behind.”

Others drew attention to the concentration of productive and decision-making authority, emphasizing stark asymmetries in the ownership and control of economic value. Participants highlighted how control over production and value appropriation is highly centralized, shaping what is produced, how resources are allocated, and whose interests are prioritized within policy processes. As Anonymous N14, a social epidemiologist, noted:

“It's capitalism because the amount of power to produce and appropriate the value of what is produced in the society is in a very small number of hands.”

Participants also situated these dynamics within longer historical trajectories, pointing to recurring moments of resistance and demands for systemic change. These moments were described as arising when the social costs of unequal wealth and power become intolerable, thereby fueling collective responses to widening inequality. Professor Trevor Hancock, a health scholar and founding leader of the Green Party, reflected:

“A free enterprise system inevitably results in very few people being extremely wealthy. And with wealth comes power—and a lot of other people being very poor and powerless . . . . Then things get so bad that the people at the bottom revolt. We see that from time to time. The French Revolution, obviously. The Russian Revolution 150 years later. It was happening on the streets of Europe in 1848.”

Across accounts, participants portrayed capital accumulation and power imbalances as organizing features of governance and policy decision making, shaping whose interests are advanced and whose needs remain unmet. Persistent inequities were articulated as arising from institutional arrangements and economic logics that normalize elite advantage and constrain redistributive possibilities. Participants commonly suggested that advancing health equity would require confronting these underlying structures rather than relying on incremental reforms.

### Theme 2: Corporate Dominance and the Marginalization of Equity Agendas

Participants consistently pointed to the dominant role of corporate actors in Canadian policymaking processes. Corporate power was widely understood as embedded within routine policy operations and institutional practices. Sixteen participants reported that policy outcomes were commonly experienced as reflecting economic interests, capital power, and prevailing ideologies, with limited responsiveness to public need or research evidence. Thirteen viewed advocacy spaces as marked by unequal access, describing policymaking arenas as more accessible to elite economic and political actors than to those advancing equity-focused agendas.

Corporate influence was described as operating through institutional advantages such as political connections, lobbying capacity, and privileged access to decision-makers, which participants viewed as narrowing the scope of grassroots participation and impeding redistributive policy initiatives. As Professor William Carroll, a political sociologist, explained:

“When we look at the fossil fuel sector and its relationship to the federal and provincial government . . . we’ve mapped out the lobbying relations and the access that Big Capital has to the major players, the major kind of deputy ministers and state managers and politicians . . . . The access big corporations have is just incredible . . . . Powerful economic forces are well-resourced . . . and it's difficult for NGOs [non-government organizations], social movements, and community groups to match that in terms of their voice. The research that we’ve done makes it clear that there's this real, very un-level playing field . . . . The corporate agenda tends to be accepted by the political leadership.”

Across participants’ accounts, policy evidence was indicated as carrying limited influence when it conflicted with dominant economic interests or ideological commitments. Scientific findings were often said to fail to gain traction when they challenged strategies designed to ensure corporate profit. This perspective reflects concerns expressed by participants regarding the marginal role of evidence in policymaking shaped by corporate power. As Hancock noted:

“Oh no, not at all. What I’ve said for years is the evidence that evidence changes policy is pretty slim . . . . We have all kinds of evidence, but is it changing policy? Hardly at all . . . . The evidence that most of the fossil fuel in Canada has to stay in the ground if we want to avoid two degrees centigrade warming doesn’t really seem to affect policy . . . . The right-wing, the Republicans, some Conservatives here, their rejection of the evidence has nothing to do with the evidence. It's really an ideology . . . . Our group thinks this way, and we are aligned with the fossil fuel industry because they’re rich and powerful . . . . The evidence on tobacco took 30, 40 years . . . and it's still not fully effective . . . . And now we have the tobacco industry going into the vaping industry.”

Participants from labor and advocacy organizations described similar dynamics within employment and labor policy, emphasizing how organized business groups mobilize substantial resources to oppose regulatory change. The backlash against *Ontario's Bill 148: Fair Workplaces, Better Jobs Act (2017)*—which raised wages, expanded paid sick leave, advanced pay equity, improved scheduling protections, and strengthened union rights—was cited as a clear example.^30^ This case was referenced to illustrate how employer groups draw on financial resources, political networks, and communications strategies to weaken legislation aimed at reducing workplace inequalities. Economist Sheila Block observed:

“The enormous effort and pushback from the Chamber of Commerce and others against . . . Bill 148 . . . . There's unequal access to resources . . . . Interests in favor of the status quo have a lot more resources than progressive groups do . . . . They can buy a lot of communications advice . . . . It's really a very unequal kind of advocacy effort between grassroots groups . . . and employer groups like the Chamber of Commerce.”

Participants working in labor and health sectors also identified campaign financing practices, “revolving doors” between government and industry—where individuals move between regulatory roles and corporate positions—and close proximity to policymakers as mechanisms reinforcing power imbalances within policy processes. As Michael Hurley, a health care union president, emphasized:

“We don’t have the same level of influence . . . . Unions in Ontario's health care sector—and the Health Coalition—are set off against powerful interests, like pharmacological companies and the private long-term care industry. These companies offer jobs and directorships to politicians and senior bureaucrats. They bankroll campaigns. They are political donors . . . . They are very powerful interests . . . more influential with the government.”

At the same time, participants underscored that corporate influence was experienced as dominant but not absolute. Several recalled specific instances in which coordinated labor and community mobilization successfully shifted policy direction, while also noting that such gains were intermittent and dependent on sustained organizing. As Anonymous N11, a labor leader, explained:

“Corporation wins 60 to 65% of the time . . . but there's an opportunity depending on the issue of the day or the catalyst behind folks trying to organize and mobilize. There's a chance for the pro-labor community and allies to work together to move the government . . . . The fight for a $15 minimum wage . . . the government didn’t just do that on their own. It was a push from organizations in the community, labor, policymakers, and researchers who said: ‘If we want a good Ontario, we need to do this.’”

Political theorist Professor Leo Panitch similarly emphasized asymmetry alongside contingency, highlighting the relational nature of class power:

“There's a vulgar way of looking at this, which is to say that business has all the power. If that were the case, you wouldn’t be able to explain why we have Medicare in Canada. Class relations reflect reciprocal power. There is [an] asymmetry. Capital has more power than labor—but because labor is necessary, that means labor has some power. And depending on how well it organizes, and with what degree of political understanding it organizes—whether labor is just selling itself as a commodity to buy more commodities or whether it's organizing . . . . That applies to any assessment of which interest groups have more power than others.”

Across accounts, participants framed corporate power as entrenched within political and institutional settings, shaping policy agendas and delimiting the range of outcomes considered feasible. These dynamics were understood to accommodate corporate capital interests within policymaking processes, influencing whose priorities gain traction and whose claims are marginalized. Health equity policy was interpreted as unfolding within these political-institutional arrangements, shaping which initiatives advance and which redistributive reforms are stalled or reversed.

### Theme 3: Neoliberal State Norms and the Narrowing of Democratic Space

Participants described state institutions and governing practices as actively shaping the reproduction of social and health inequities. All 23 participants characterized Canadian policymaking as deeply seated within a political–economic context aligned with capital agendas, with insufficient attention to the concerns of workers and communities. Participants identified gaps between democratic principles and everyday encounters with political participation, which were commonly experienced as uneven, selectively accessible, and difficult to navigate.

Access to decision making was widely portrayed as stratified, with well-connected actors benefitting from professionalized advocacy channels that participants viewed as reinforcing exclusion. Historically subordinated groups were reported to face greater barriers to influencing policy. Dr Kwame McKenzie, a physician and health equity policy advisor, explained:

“Part of the problem is that there are competing groups, and the fact that groups competing with each other makes it difficult for change to happen because they get played off against each other. But organized groups with a constituency that will vote on their issue have a pretty loud say . . . . People who know how to get in front of the right people in government, and who know how to produce a pitch that's a win-win situation . . . are the people who are going to be most effective.”

Public consultation processes were depicted as procedural exercises with little bearing on final policy decisions. Participants interpreted these practices as perpetuating existing hierarchies of influence and narrowing opportunities for substantive participation. As Anonymous N11, a health care union leader, noted:

“The government is . . . elected by the people, but certain governments take the position that they don’t really need to listen to the people . . . . We’ve heard backdoor dealings with corporations and multinational organizations who’ve been part of the decision making, who’ve been at the table, getting their voice through legislation. But the frontline worker—the average member of society—their voice, isn’t reflected. You don’t get a democracy. People should have a voice, and people should be able to voice their concerns.”

Several participants questioned dominant accounts of inclusive, multi-stakeholder governance, seeing them as misaligned with observed policymaking practices. Governments were portrayed as selectively responsive, with greater accommodation extended to business and corporate actors. John Clarke observed:

“That's a very intriguing question because of the myth that government put forward. And Liberals are generally better at doing this, although the Tories played the same game, just not quite as well. But they try to suggest that governments are sort of wise King Solomons who listen to everybody's point of view and then [decide] in the best interest of most people. I’ve never found that to be the case. The notion that all stakeholders come to the table equally . . . respected and listened to by the government is . . . a myth. The sham of consultation is a shame. I can’t say that I’ve seen any Ontario government—or any government I’ve ever experienced anywhere in both countries that I’ve lived in [Canada and the UK]—operate as anything other than essentially an executive committee for rich people . . . . The Chamber of Commerce and the representatives of big business come to the table . . . . They’re the ones . . . accommodated.”

Participants also raised concerns about the limitations of electoral politics. Governing parties were located within a narrow ideological range, with political competition centered on managing dissent rather than enabling structural change. Professor William Carroll reflected:

“They’re both very corporate . . . . At the federal level, what we have is this kind of revolving door between the Liberals and the Tories, and one comes into power. The Harper government was especially bad, but the Trudeau government isn’t much different . . . . In terms of really addressing issues of class, gender, and race inequality and how they manifest themselves in people's lives, I don’t think either of those parties has very strong commitments to change. They’re mainly interested in managing dissent and trying to secure enough consent that there isn’t any political crisis . . . . Business as usual; that's their primary goal politically.”

Neoliberal policy trajectories—marked by privatization, austerity, deregulation, and labor market flexibilization—were presented as extending across successive governments and accumulating over time. Participants linked these trajectories with declining housing security, weakened employment protections, reduced income supports, and growing pressure on public health services. Health sector reforms were frequently cited as concrete manifestations of this restructuring. As Natalie Mehra, Executive Director of the Ontario Health Coalition, explained:

“We see a very severe erosion of health care services, particularly targeted to the elderly. Within the health care workforce, we have seen a downloading of patients from fully public modes of care that provide full care, like hospitals, to privatized modes of care, like long-term care and home care. Similarly, a downloading and deregulation of the workforce from RNs [registered nurses] to RPNs, [registered practical nurses] and now to PSWs [personal support workers]. So, much heavier workloads with much lower pay and working conditions. And a very significant racialized workforce that has taken these heavier care patients with less funding, less resources, less support, and less training . . . . There have also been very dramatic cuts to government funding.”

Several participants interpreted these developments as a deliberate political project designed to weaken collective capacity and reshape labor markets to facilitate capital accumulation. Political economist Dr Greg Albo described neoliberalism as a policy regime oriented toward labor market flexibilization and capital concentration:

“The policy regime of neoliberalism is designed to force flexibilization and allow the concentration of capital. Flexibilization was a code word for lowering the bottom end of the labor market, the welfare support, the housing support, and so on, and weakening union power so you’d have more dispersion in wage outcomes and more flexibility in the labor market. Its foundation was to generate inequalities at both ends. This was the policy in capitalist governments—of both the parties of the right and parties of the centre. It encompassed the Conservative parties. It encompassed the Liberal parties. And steadily, it covered the parties of the centre-left—the social democratic parties.”

Participants further highlighted institutional constraints woven into the architecture of the capitalist state, which they viewed as structurally bounding democratic influence over core political and economic arrangements. Albo elaborated:

“This is really a question of the capitalist state and capitalist power. The possibilities of influencing the state are limited under capitalism because a capitalist state is structured to limit democracy through a representative form—meaning elected officials of a most minimal kind deliberate about the tactics over the reproduction of the existing society. And that existing society includes the forms of production, the forms of ownership, the forms of private property, and so forth. Everything that would challenge those structures is kind of put to the side or marginalized.”

These constraints were also understood to reproduce political inequality through systems of representation and mobilization. Participants underscored how class advantage is carried through party competition, access to resources, and media visibility. As Albo noted:

“The political system from the beginning establishes a series of blockages [to] noncapitalist solutions to any social problem. There are also the blockages that go along with inequalities. The capitalist political systems divide into parties of the business classes, the property classes, parties of the middle classes, and parties of the working classes . . . . And they’re contesting over different policies and maybe even over capitalism itself. But the system is structured particularly to support the parties of the capitalist class. That is, the systems of representation, the amount of money in politics, the access to media, and so on . . . . So, there's a structural inequality established in the system of political representation and the systems of political mobilization. So, the terrain of the state is in itself structured to be a terrain that reproduces that class relationship and, therefore, the inequalities of that society, and so forth.”

Across accounts, participants presented state governance as organized through institutional arrangements that privilege existing political–economic relations and delimit the scope of democratic influence. Democratic participation was cast as procedural and narrowly bounded, with restricted capacity to shape redistributive outcomes or challenge underlying class relations. Taken together, these conditions were viewed as defining the constrained political terrain within which health equity policy is pursued.

### Theme 4: Civil Society Fragmentation and the Weakening of Collective Influence

All 23 participants identified fragmentation within civil society as undermining the capacity to advance redistributive policy and address health inequities. Across interviews, divisions among labor organizations, advocacy groups, and social movements were viewed as diminishing collective leverage and narrowing opportunities for sustained challenges to concentrated power.

The erosion of labor solidarity was a recurring focus. Participants pointed to concessionary bargaining, sectoral competition, and internal divisions as dynamics that weaken union influence and heighten exposure to employer pressure and policy retrenchment, particularly in contexts shaped by economic restructuring. Dr Toba Bryant, a health policy expert, reflected:

“Some unions have engaged in concessionary bargaining, thinking that aligning themselves more closely with a corporate employer will save jobs and protect their workers—but it doesn’t. We saw that happen in Oshawa with the GM [General Motors] plant.”

Participants further highlighted weak coordination among advocacy organizations working on related issues, resulting in parallel equity-oriented policy actions that remain disconnected. This fragmentation was regarded as diluting collective impact, even when campaigns addressed shared redistributive concerns. Jonah Gindin, a health care union leader, explained:

“You can have different unions . . . who have completely separate campaigns . . . ultimately, in some cases—they’re identical issues . . . . A union with health care workers had . . . a campaign around combating the profit-taking in long-term care . . . yet there's little to no coordination . . . . Campaigns around public health care are treated as completely separate issues . . . and yet . . . they’re about redistribution of wealth, redistribution of power, and expansion of public services and rights to people.”

Fragmentation was articulated as dispersing political constituencies across narrow issue areas, weakening the capacity to mobilize sustained collective pressure. Participants observed how siloed organizing diffuses political voice and fragments collective leverage across competing interests. Dr Kwame McKenzie emphasized:

“If you can silo different interest groups, you can essentially make them ineffective because none of them have a big enough constituency to bring down governments. When the unions were strong, and when I was in the UK, they could bring down the government. But at the moment, we have loads of siloed, smaller pressure groups who are not really articulating. You’ve got people who are interested in housing. People are interested in health. People are interested in race and racism. People are interested in gender. People are interested in trans issues. People are interested in city issues. So, it goes on. The more people focus on smaller siloed issues, the less effective they are overall . . . . They all get happy with getting crumbs from the table—and then they all forget it's actually their table . . . . We need a rainbow coalition.”

Several participants traced these patterns to historical strategies within labor movements that secured sector-specific gains without extending them universally, a trajectory they associated with growing vulnerability to isolation and division over time. Sam Gindin, a labor activist, reflected:

“In the ‘50 s and ‘60 s . . . unionized workers were able to win good programs for themselves: pensions, health care insurance, pharma care, dental care. But they won it for themselves. It wasn’t a universal program. The problem with that is . . . as circumstances changed, the workers who won things were attacked. And they became isolated—they had it, other people didn’t . . . . When you don’t win it for everybody, it's always dangerous. It's always divided . . . . There's a problem among workers themselves for not having a class perspective.”

Participants emphasized that even well-organized movements act within structural conditions that shape the sustainability of collective gains. Advances secured through collective action were characterized as partial and vulnerable to reversal, particularly where profit-oriented priorities remain dominant. Gindin elaborated:

“The bigger problem is that these are political questions. If you’ve got an unequal society, you’re going to have inequalities in politics . . . . Power . . . it's always the fundamental question . . . . Even if you’re strong, if you’re operating under capitalism, there will always be limits. Because capitalism—it's not there to improve your health; it's there to make profits. So, you’re always fighting the logic of the system, which means that you’re always defensive, even when you’re making some gains.”

Across accounts, participants identified civil society fragmentation as a constraint on collective influence that operates in tandem with institutional power, shaping the limits of equity advocacy. Divisions within civil society were understood to diffuse political leverage and impede sustained challenges to concentrated power. Advancing health equity was consistently linked to the formation and strengthening of collective capacity through broader coalitions organized around shared redistributive priorities.

## Discussion

The Discussion section presents interpretations of the four empirical themes by situating them within broader political-economic patterns shaping policymaking in Canada, reorganizing them into analytically distinct yet interrelated structural dynamics. Building on the Findings section, the analysis draws on critical political economy scholarship and related work on power and governance to examine the conditions under which equity-oriented reform is pursued and constrained. Whereas the Findings section shows the documented empirical patterns, the Discussion section considers how these dynamics illuminate the organization, distribution, and reproduction of power across economic, political, and institutional domains.

Rather than treating the four themes as discrete or sequential factors, the analysis conceptualizes them as interlocking dynamics that operate together within capitalist policymaking contexts. These dynamics are examined as recurring configurations in which institutional arrangements prioritize accumulation, corporate actors exert sustained influence over decision-making, neoliberal governance narrows the range of politically plausible policy responses, and fragmented collective organization limits countervailing pressure. Taken together, they help explain how constraints on health equity reform are reproduced across policy domains, even where formal commitments to equity are present.

Table 2 synthesizes these dynamics within a critical political economy interpretive framework. The table is not intended as a typology of causal mechanisms, but as an analytic map illustrating how these dynamics diverge from assumptions commonly associated with pluralist models of policymaking—particularly expectations of equal access, balanced responsiveness, and democratic inclusion. Pluralist concepts are engaged here as empirical reference points, clarifying how policymaking processes are structured by enduring power asymmetries rather than neutral competition among interests.

**Table 2.** Mapping of Empirical Dynamics to Interpretive Framework and Structural Implications.

Dynamic	Interpretive Orientation	Structural Implication
Structural–Institutional Power and Democratic Constraints	Critical political economy; diagnostic reference to pluralist assumptions	Governance arrangements embed enduring power asymmetries that delimit the scope of redistributive reform.
The Institutionalization of Corporate Power in Policymaking	Political economy of corporate–state integration	Policymaking processes are structured around market priorities, shaping which equity initiatives are considered feasible.
Neoliberal Governance and the Narrowing of Policy Horizons	Political economy of neoliberalism; diagnostic reference to pluralist assumptions	Policy agendas are constrained through fiscal and market logics that circumscribe transformative options.
Civil Society Fragmentation and the Erosion of Collective Agency	Political economy of labor, advocacy, and state relations	Fragmented organizational landscapes weaken sustained countervailing influence within policymaking processes.

### Structural–Institutional Power and Democratic Constraints

Power imbalances shaping health equity policymaking in Canada are better understood as closely tied to the institutional features of capitalist governance. Policymaking unfolds within structural configurations that privilege capital, consolidate elite influence, and circumscribe redistributive agendas. From this vantage point, the analysis raises fundamental challenges to pluralist assumptions that policy arenas are broadly accessible, equally responsive, or characterized by balanced competition among competing interests, instead pointing to persistent asymmetries that operate through governance processes themselves.

These dynamics align closely with critical political economy scholarship, which conceptualizes health policy as organized through interlocking economic, political, ideological, institutional, and societal forces that systematically reproduce inequality.^1,31-33^ From this perspective, capital and state power are central to shaping the distribution of resources and structuring access to health-promoting conditions and services. In capitalist societies such as Canada, the institutional architecture of the state is predisposed toward capital accumulation over public need,^1,3,4,6-8,31-33^ delimiting the scope of redistributive reform and embedding inequality within policymaking itself.

Contemporary economic data reinforce this structural analysis. In 2024, corporate billionaires amassed US$2.8 trillion—approximately US$8 billion per day—while global poverty levels stagnated. This wealth accumulation was enabled by monopoly control, speculative finance, political capture, and inheritance.^34^ The Global North's top 1% extracted an estimated US$42 million per hour from the Global South, sustaining patterns of neocolonial accumulation that operate across global political–economic relations.^34^ In Canada, billionaire wealth increased by CAD$113 billion as 3.8 million people lived in poverty; 61% of this wealth was unearned, and the top 1% extracted CAD$2.66 billion from the Global South.^34^ Such extreme concentration of wealth—a well-established upstream driver of health inequities—reflects the predictable outcome of a capitalist political economy structured to consolidate economic and political power.

The analysis further highlights how mechanisms such as stakeholder consultations often simulate deliberation while legitimizing predetermined outcomes, producing the appearance of inclusion without altering underlying power relations. These processes function less to democratize decision-making than to stabilize existing institutional arrangements. This challenges classical pluralist claims that procedural inclusion alone confers democratic legitimacy.^10-12^ Prior scholarship similarly documents how redistributive health equity policy proposals advanced by civil society are routinely dismissed as unrealistic or fiscally untenable,^12,35,36^ reinforcing technocratic approaches grounded in managerialism and incrementalism.^13-17^ Inclusion absent decision-making authority thus operates less as democratization than as institutionalized tokenism.

The persistence of these asymmetries undermines the democratic quality of policymaking processes. Advocacy that does not confront the structural entrenchment of capital remains vulnerable to co-optation, reproducing the very systems it seeks to contest.^35,36^ While technocratic adjustments and incremental reforms may generate limited tactical gains, they do not alter the underlying power through which inequities operate.^37-40^ Advancing health equity therefore requires a shift from transactional engagement toward transformative political strategies that challenge the institutional foundations of the capitalist state and reclaim public institutions as vehicles for democratic redistribution. Absent such structural change, health inequities and democratic erosion are likely to persist.

### The Institutionalization of Corporate Power in Policymaking

Corporate dominance in Canadian policymaking is better understood as institutionalized rather than incidental. Corporate actors—particularly within fossil fuel, health care, and labor sectors—function as embedded policy actors rather than external influencers, often positioned as primary *policy entrepreneurs*.^12^ Through privileged access to regulatory bodies, political leadership, and consultative forums, these actors shape policy agendas to advance capital interests while narrowing the scope of health equity-oriented reform. Lobbying networks, regulatory appointments, and revolving-door relationships blur boundaries between governance and private gain, producing institutional arrangements in which public interest claims are structurally subordinated.

This pattern aligns with evidence from the Corporate Mapping Project, which documents how fossil fuel corporations maintain dense, interlocking networks of influence within the Canadian state.^41^ Such networks enable corporate actors to steer health and environmental policy toward market imperatives,^41-44^ illustrating forms of corporate–state integration in which governance operates in alignment with private accumulation rather than public or planetary well-being. In 2024 alone, fossil fuel lobbyists registered more than 1100 lobbying activities in Canada.^43^ These same corporations—whose operations disproportionately harm Indigenous, racialized, and low-income communities—retain decisive influence over policies ostensibly designed to mitigate such harms.^41-44^ This degree of integration reflects what is commonly described as corporate capture, wherein public institutions are repurposed to advance corporate profit and political power.

The consolidation of corporate power sustains environmental and health injustice by concentrating low-income and racialized populations near polluting industries and exposing them to unsafe air, water, and hazardous land-use decisions. Across sectors, capitalist expansion has produced sacrifice zones in which environmental and health burdens fall most heavily on populations with the least economic and political power.^33^ These patterns reflect a political economy that prioritizes capital accumulation over human and planetary health, shaping health equity outcomes through prevailing power relations rather than through evidence of harm or population need.

Labor and employment policy reflects similar dynamics. The dismantling of progressive provisions under Ontario's Bill 148 through Bill 47—which froze minimum wage increases, repealed equal pay protections, weakened scheduling and union rights, and reduced paid leave^45^ —illustrates how labor protections can be swiftly reversed when they threaten capital interests. These reversals demonstrate that the durability of health-promoting policy depends less on evidence than on the balance of power shaping political decision-making, and that the same institutional forces curtailing labor rights also obstruct health equity reform. Capitalist state institutions consistently operate to protect economic power rather than collective well-being.

At the same time, the analysis underscores that corporate power is formidable but not immutable. Campaigns such as the Fight for $15 illustrate how convergence among labor organizations, advocacy groups, social movements, and progressive policy actors can disrupt dominant agendas and secure tangible gains. Yet such victories were widely understood as narrow, contingent, and vulnerable to reversal.^12^ Absent sustained countervailing power, corporate dominance is continually reproduced through institutional routines, administrative practices, and ideological narratives. Power remains relational—and therefore contestable—but meaningful shifts require strategies that systematically advantage workers, communities, and marginalized populations within policymaking processes.

### Neoliberal Governance and the Narrowing of Policy Horizons

Neoliberal governance narrows the range of equity-oriented policy options in Canada. Neoliberalism operates as a governing rationality embedded within state institutions, prioritizing market efficiency, privatization, deregulation, and fiscal restraint as default policy logics. Within this framework, health equity mandates are routinely subordinated to managerial and budgetary imperatives, shifting policymaking away from redistribution and public provision toward commercialization, cost containment, and market-aligned regulation.

Ontario'*s Bill 60, Your Health Act (2023)* exemplifies these dynamics. Framed as an efficiency measure, it expands the role of for-profit clinics in delivering publicly funded services,^46^ shifting work from public to private settings, driving workforce de-skilling, and lowering workplace standards.^33^ Nationally, similar reforms privilege cost containment over quality of care, private provision over public infrastructure, and individual responsibility over collective solidarity.^1,4,6,7,33^ The termination of public housing programs, the dismantling of social services, and chronic underfunding have further deepened inequities, particularly for racialized, Indigenous, and working-class communities.^1-7,47-50^ In this way, neoliberal logics delimit the scope of reform, relegating health equity objectives to the margins of policy considerations.

The distributional consequences of neoliberal restructuring are stark and cumulative. Between 1990 and 2010, incomes for the top 0.01% increased by 145%, while earnings for most working-class households stagnated,^51^ entrenching widening income polarization. This divergence has been reinforced by the systemic erosion of labor protections—reflected in declining union density, weakened collective bargaining, and the expansion of precarious employment—producing lasting forms of economic insecurity.^33^ By 2016, Canada's 87 wealthiest families held as much wealth as 12 million lower-income residents—an amount roughly equivalent to the combined assets of those living in New Brunswick, Newfoundland and Labrador, and Prince Edward Island.^52^ Such concentration is not incidental but reproduced through differential saving capacity, intergenerational wealth transfers, and a tax regime that privileges capital gains while enabling avoidance.^52^

These patterns reinforce Navarro's observation that neoliberal states prioritize capital accumulation over social protection, narrowing the political space available for health equity agendas.^53^ Precarious work, widening income gaps, and persistent health inequities thus appear not as policy anomalies but as recurring features of neoliberal political economy.^53,54^ In Canada, major political parties remain broadly aligned with these priorities,^55-57^ preserving a policymaking apparatus shaped more by capital imperatives than democratic inclusion.^57-59^ While equity rhetoric persists,^3^ structural logics that subordinate care to capital continue to constrain implementation.

Within this configuration, the neoliberal state emerges less as a neutral arbiter among competing interests than as a political formation oriented toward safeguarding accumulation. Pluralist assumptions therefore operate less as empirical descriptions of democratic contestation than as legitimating narratives for market-aligned policy outcomes.^55-59^ Public engagement processes frequently project consensus and participation while obscuring the structural incompatibility between neoliberal governance and health equity objectives. Neoliberalism functions materially by channelling public resources, authority, and risk toward private accumulation, and ideologically by narrowing the range of politically acceptable responses and reframing structural injustice as technical inefficiency or individual responsibility.^33,53,54^ These dynamics constrict the political space for redistributive demands,^3,33,53,54,57,60^ while participation mechanisms deliver symbolic inclusion without redistributing decision-making power. Absent transformation of these structural–institutional logics, health equity policy remains tethered to profit imperatives that set the outer limits of feasible reform, rather than organized around collective need.

### Civil Society Fragmentation and the Erosion of Collective Agency

Fragmentation within civil society has substantially weakened its capacity to sustain collective pressure for redistributive policy. Once a countervailing force to state and corporate power, civil society has been reshaped under neoliberal governance into a more defensive and institutionally constrained formation. Sectoral divisions, professional segmentation, and the absence of sustained cross-sector coordination have diminished collective capacity, leaving reform efforts vulnerable to co-optation and reversal.

From a classical pluralist perspective, organized interests are assumed to influence policy through mobilization, bargaining, and negotiation within broadly accessible political arenas.^10-12^ This assumption contrasts with analyses demonstrating that civil society operates within political terrains structurally organized to privilege dominant capital interests.^13-17^ Rather than conditions of equal access or balanced contestations, policymaking is structured as unevenly resourced and selectively responsive, with elite actors enjoying disproportionate influence. Under these conditions, health equity policymaking emerges less as a neutral process of interest aggregation than as a contested terrain shaped by capital and class power; advocacy that does not directly confront these structural relations remains reactive, fragmented, and limited in its capacity to secure redistributive change.

While existing research shows that strong labor institutions can improve health equity outcomes,^60-64^ their transformative potential is constrained in the absence of cross-sectoral alliances and a shared vision for structural change. Historical patterns illustrate this limitation. Post-war labor victories—such as employer-based health benefits—were secured through narrow occupational negotiations rather than universalist demands, entrenching hierarchies within the working class.^33,57^ These arrangements generated uneven protections and weakened broader class solidarity. Today, occupational segmentation and professional differentiation continue to inhibit collective alignment, undermining coordinated responses to privatization, labor rollback, and health system erosion.

The weakening of civil society's political influence reflects not only capital dominance and state coercion,^65,66^ but also internal disunity, competing strategic orientations, and short-term advocacy approaches. Neoliberal restructuring has intensified these vulnerabilities through declining union density, curtailed bargaining rights, and a diminished infrastructure for collective resistance.^33,57,62,63^ Health, labor, and community movements often operate in isolation, a fragmentation reinforced by competing mandates and the absence of a unifying political-economic framework capable of sustaining long-term collective action.

At the same time, the analysis indicates that the potential for collective agency persists. Historical moments of convergence—most notably the coalitions that secured Canada's publicly funded health care system—demonstrate that broad-based alliances can challenge entrenched power when organized around shared material interests and political clarity. Achieving health equity therefore requires structural transformation grounded in sustained coalition-building, ideological coherence, and a unifying vision capable of mobilizing diverse constituencies. Without reconstituting civil society as a strategically oriented countervailing force, the alliance between capital and the state will continue to operate as a defining constraint on economic and political possibility.

## Implications and Conclusions

### Implications for Power and Health Equity Policymaking

Efforts to advance health equity—whether led by researchers, advocates, workers, or community organizers—are shaped and constrained by political and economic structures that centralize power, suppress countervailing claims, and limit collective mobilization for structural change.

Across the analysis health equity emerges not as a technical challenge of evidence translation or a procedural problem of inclusion. Rather, it is a political and class struggle over the institutions and social relations that govern access to health, care, and the conditions that sustain life. Addressing entrenched inequities therefore necessitates strategies oriented toward redistributing resources, democratizing decision making, and building collective power through sustained political engagement and health activism.

Drawing on in-depth interviews with equity-oriented policy actors, this study demonstrates how capital accumulation, institutionalized corporate influence, neoliberal governance norms, and fragmented civil society converge to constrain redistributive reform in Canada. While pluralism remains useful for descriptively mapping policy actors and institutional arenas, the analysis indicates that policymaking environments are structured by enduring capital and power asymmetries rather than balanced contestation. Within these conditions, economic and political elites exert disproportionate influence, while health equity advocates and community-based organizations face systematic constraints within the same decision-making spaces.

Interpreted through a critical political economy lens, the analysis further indicates that health inequities are not the result of policy oversight, weak evidence uptake, or episodic political failure. Instead, they reflect predictable outcomes of political–economic arrangements that privilege market-aligned interests over redistributive demands. Capital–state alliances shape employment, income, housing, food systems, health services, and ecological conditions,^1,4,6,7,31-33,41-44,67^ while labor unions and grassroots movements operate from structurally disadvantaged positions within these same policy arenas. In this context, institutionalist explanations that emphasize capacity deficits, evidence gaps,^35,68-71^ or insufficient political will,^36,72,73^ obscure how structural constraints shape political feasibility itself.^23,24,31-33,53-60,74-77^ Neoliberal governance further narrows the space for redistributive reform,^31-33,53-57,78^ exposing enduring tensions between capitalist democracy and the realization of health equity.

Taken together, these findings underscore the limits of pluralist assumptions that health equity policymaking arenas are broadly open, equally accessible, or democratically responsive. A critical political economy approach is therefore essential for understanding how health inequities are produced and sustained—not as technical failures of policy design or implementation, but as outcomes of institutionalized power relations embedded within capitalist governance.

### Reframing Health Theory and Advocacy

Institutional frameworks such as the *World Report on Social Determinants of Health Equity*^79^ and the Health in All Policies (HiAP) have played an important role in advancing upstream, cross-sectoral approaches to health equity.^36^ While broadly aligned with holistic public health principles, these frameworks often stop short of directly confronting the state–capital nexus that structures policy priorities and constrains redistributive action. This limitation does not reflect an absence of political awareness. Contrary to depoliticized readings of SDH discourse,^24^ these frameworks do engage questions of power and governance—but in ways that remain bounded by prevailing institutional and economic logics.

In practice, mainstream SDH and the HiAP frameworks frequently conceptualize governance as neutral, technocratic, and broadly inclusive, rendering them vulnerable to policy drift, dilution, and capture.^36-40^ Where capital accumulation and structural power relations remain insufficiently examined, equity-oriented initiatives tend to produce incremental reforms that leave the foundations of inequality intact. Although such reform may yield partial and uneven gains,^36-40^ their effects are constrained by market imperatives and the preservation of existing state-capital relations. The persistence of health inequities across class, race, Indigeneity, gender, and geography therefore reflects the limits of approaches that accommodate—rather than contest—the political-economic conditions under which policy is made.

The analysis underscores that improved evidence, expanded consultation, and administrative refinement are insufficient on their own to overcome the structural conditions that generate health inequities. Rather than failures of evidence or governance capacity, these limits reflect entrenched distribution of power and resources. Transformative change requires sustained political struggle oriented toward the redistribution of wealth, power, and decision-making authority. Effective health equity advocacy must therefore be grounded in democratic and redistributive justice—mobilizing labor power, building durable cross-sectoral coalitions, and directly confronting the structural alignment between state institutions and capital interests that delimit policy possibility.^3,33,57^

Canada's experience mirrors patterns observed across liberal welfare states, where corporate dominance, institutional bias, and civil society fragmentation shape policy trajectories.^33,55,57,60^ Health equity is thus better understood as a question of structural power, with social and ecological determinants of health emerging from the political and economic systems that govern their production, distribution, and consumption.

### Delimitations and Limitations

This study applies a critical political economy framework to examine how Canadian health equity policymaking reflects and reproduces entrenched power asymmetries. While this approach is well suited to analyzing interlocking forms of structural violence—including colonialism, neocolonialism, racism, sexism, and ableism^2,7,29,49,50^—the analysis deliberately centers political-economic dynamics of capital accumulation, state power, and institutional constraint. These dimensions are therefore examined in depth, while other axes of oppression are engaged selectively rather than exhaustively. Future research should extend this work through intersectional, decolonial, and feminist analyses to more fully examine how these systems intersect to produce and sustain health inequities.

The analysis draws on interviews with 23 Canadian policy scholars, advocates, and activists. While this sample provides rich insight into how equity-oriented policy actors interpret structural drivers of health inequities, it does not capture the full diversity of regional, sectoral, or lived experience across Canada. Future research should more centrally foreground Indigenous, racialized, gender-diverse, and community-based perspectives, particularly those most directly affected by structural exclusion and material harm.

As a racialized immigrant with experience in factory work, care work, frontline nursing, and academia, my analytic perspective is shaped by sustained engagement with structurally unequal institutions. This positionality informs a reflexive approach to how power, class, and institutional location contour both health inequities and the politics of equity-oriented policymaking.

### Conclusion and Future Directions

Structural health inequities in Canada persist—and in many cases have intensified—despite decades of equity-oriented policy commitments. This study shows that these outcomes are rooted in the organization of capitalist governance, in which corporate capture, elite political consensus, and fragmented civil society resistance consistently limit the possibility for transformative change.

Grounded in critical political economy, the analysis identifies four mutually reinforcing dynamics shaping the production and persistence of health inequities: concentrated economic and political power; the institutionalization of corporate influence in policymaking; neoliberal state structures that constrain reditributive policy action; and civil society fragmentation that weakens oppositional capacity. While these dynamics resonate with long-standing critiques of pluralist governance, the findings clarify how political-economic structures decisively shape the boundaries within which health equity policy action becomes possible.

This article advances health equity scholarship in three key ways. First, it provides qualitative empirical evidence of how equity-oriented policy actors interpret and navigate the political–economic foundations of health inequities in Canada. Second, it anchors critical political economy theory in the lived and professional experience of equity-oriented policymaking. Third, it identifies moments of strategic disruption in which coordinated action across labor, health, and community sectors has challenged entrenched power relations, demonstrating that these structures—while formidable—remain contingent and contestable.

The implications of these findings are substantive. Health equity theory must engage the capitalist state and capitalist power as central determinants shaping health outcomes, rather than treating health inequities as policy deviations or implementation failures. Health advocacy and practice must move beyond technocratic intervention toward sustained, cross-sectoral coalitions capable of contesting institutionalized power and reshaping the conditions under which policy decisions are produced.

Incremental reform alone cannot resolve structural health inequities. Advancing health equity requires redistribution of wealth and power, democratization of political and economic institutions, and the renewal of collective political agency from below. Health justice becomes possible within a social order oriented toward democratic redistribution, ecological regeneration, and collective care—one that treats social well-being not as a market outcome, but as a shared political responsibility.

## Data Availability

This study is part of the author's doctoral dissertation submitted to York University.

## References

[1] Armstrong P Armstrong H Coburn D . Unhealthy Times: Political Economy Perspectives on Health and Care. Oxford University Press; 2001.

[2] Hankivsky O Grace D Hunting G , et al. An intersectionality-based policy analysis framework: Critical reflections on a methodology for advancing equity. Int J Equity Health. 2014;13(1):119. doi:https://doi.org/10.1186/s12939-014-0119-x 10.1186/s12939-014-0119-x PMC4271465 25492385

[3] Borras AM . Toward an intersectional approach to health justice. Int J Health Serv. 2021;51(2):206-225. doi:10.1177/0020731420981857 33356774

[4] McGibbon E . The Politics of Social, Ecological, and Structural Determinants of Health in Canada. Canadian Scholars; 2024.

[5] Gunn V O’Campo P Muntaner C . Exploring indigenous health and employment: Methods to capture diversity and intersecting factors. Am J Public Health. 2025;115(5):634-636. doi:10.2105/AJPH.2025.308030 PMC11983042 40203263

[6] Bryant T , ed. Handbook on the Social Determinants of Health. Edward Elgar Publishing; 2025.

[7] Raphael D , ed. Social Determinants of Health: Canadian Perspectives. 4th ed. Canadian Scholars Press; 2025.

[8] Muntaner C Navarro V . Conclusion: Political, economic, and cultural determinants of population health—a research agenda. In: Navarro V Muntaner C , eds. Political and Economic Determinants of Population Health and Well-Being: Controversies and Developments. Baywood Publishing Company; 2004:551-556.

[9] Navarro V Muntaner C Borrell C , et al. Politics and health outcomes. Lancet. 2006;368(9540):1033-1037. doi: 10.1016/S0140-6736(06)69341-0 16980120

[10] Lindblom CE . The science of “muddling through”. Publ Adm Rev. 1959;19(2):79-88. https://www.jstor.org/stable/973677

[11] Dahl RA . Who Governs? Power and Democracy in an American City. Yale University Press; 1961.

[12] Kingdon JW . Agendas, Alternatives, and Public Policies. 2nd ed. Pearson; 2014.

[13] Mills CW . The Power Elite. Oxford University Press; 1956.

[14] Cawson A . Pluralism, corporatism and the role of the state. Gov Oppos. 1978;13(2):178-198. doi:10.1111/j.1477-7053.1978.tb00542.x

[15] Lindblom CE . The market as prison. J Polit. 1982;44(2):324-336. doi:10.2307/2130588

[16] McFarland AS . Neopluralism. Annu Rev Polit Sci. 2007;10(1):45-66. doi:https://doi.org/10.1146/annurev.polisci.10.072005.152119

[17] Howlett M Ramesh M Perl A . Studying Public Policy: Policy Cycles and Policy Subsystems. 4th ed. Oxford University Press; 2020.

[18] Maani N Collin J Friel S , et al. Bringing the commercial determinants of health out of the shadows: A review of how the commercial determinants are represented in conceptual frameworks. Eur J Public Health. 2020;30(4):660-664. doi:10.1093/eurpub/ckz197 PMC7445044 31953933

[19] Maani N Petticrew M Galea S . The Commercial Determinants of Health. Oxford University Press; 2023.

[20] Bambra C Fox D Scott-Samuel A . Towards a politics of health. Health Promot Int. 2005;20(2):187-193. doi:10.1093/heapro/dah608 15722364

[21] Ottersen OP Dasgupta J Blouin C , et al. The political origins of health inequity: Prospects for change. Lancet. 2014;383(9917):630-667. doi:10.1016/S0140-6736(13)62407-1 24524782

[22] Kickbusch I . The political determinants of health—10 years on. Br Med J. 2015;350:h81. doi:10.1136/bmj.h81 25569203

[23] Gore R Parker R . Analysing power and politics in health policies and systems. Glob Public Health. 2019;14(4):481-488. doi:10.1080/17441692.2019.1575446 30773135

[24] Karatekin C Gresham B Barnes AJ Corcoran F Kritzik R Mason SM . Re-politicizing the WHO’s social determinants of health framework. Health Promot Int. 2024;39(5):daae122. doi:10.1093/heapro/daae122 PMC12099297 39322424

[25] Braun V Clarke V . Using thematic analysis in psychology. Qual Res Psychol. 2006;3(2):77-101. doi:10.1191/1478088706qp063oa

[26] Nowell LS Norris JM White DE Moules NJ . Thematic analysis: Striving to meet the trustworthiness criteria. Int J Qual Methods. 2017;16(1):1-13.doi:https://doi.org/10.1177/1609406917733847

[27] Brophy JT Keith MM Hurley M Slatin C . Running on empty: Ontario hospital workers’ mental health and well-being deteriorating under austerity-driven system. New Solut. 2024;34(3):182-197.doi:https://doi.org/10.1177/10482911241267347 10.1177/10482911241267347 39113552

[28] Dassieu L Pagé MG Lacasse A , et al. Chronic pain experience and health inequities during the COVID-19 pandemic in Canada: Qualitative findings from the chronic pain & COVID-19 pan-Canadian study. Int J Equity Health. 2021;20(1):147. doi:10.1186/s12939-021-01496-1 PMC8220113 34162393

[29] Pilarinos A Field S Vasarhelyi K , et al. A qualitative exploration of indigenous patients’ experiences of racism and perspectives on improving cultural safety within health care. CMAJ Open. 2023;11(3):E404-E410. doi:10.9778/cmajo.20220135 PMC10158754 37130609

[30] Legislative Assembly of Ontario. *Bill 148, Fair Workplaces, Better Jobs Act*, 2017. S.O. 2017, c. 22.Chapter Published November 2017. Accessed August 10, 2025. https://www.ola.org/sites/default/files/node-files/bill/document/pdf/2017/2017-11/bill—text-41-2-en-b148ra_e.pdf.

[31] Lynch J . The political economy of health: Bringing political science in. Annu Rev Polit Sci. 2023;26(1):389-410. doi:10.1146/annurev-polisci-051120-103015

[32] Primrose D Loeppky RD Chang R , eds. The Routledge Handbook of the Political Economy of Health and Health Care. Taylor & Francis; 2024.

[33] Borras AM . Health and Health Care Inequities: A Critical Political Economy Perspective. Fernwood Publishing; 2025.

[34] Oxfam Canada. Billionaire wealth rises $2.8T in 2024, poverty unchanged since 1990. Published January 20, 2025. Accessed May 10, 2025. https://www.oxfam.ca/news/billionaire-wealth-surges-by-2-8-trillion-in-2024-three-times-faster-than-the-year-before-while-the-number-of-people-living-in-poverty-has-barely-changed-since-1990/.

[35] Smith KE . Beyond Evidence-Based Policy in Public Health: The Interplay of Ideas. Springer; 2013.

[36] Tonelli M Tang KC Forest PG . Canada Needs a “health in all policies” action plan now. CMAJ. 2020;192(3):E61-E67. doi:10.1503/cmaj.190517 PMC6970602 31959656

[37] Shankardass K Muntaner C Kokkinen L , et al. The implementation of health in all policies initiatives: A systems framework for government action. Health Res Policy Syst. 2018;16(1):26. doi:https://doi.org/10.1186/s12961-018-0295-z 10.1186/s12961-018-0295-z PMC5856219 29544496

[38] Such E Smith K Woods HB Meier P . Governance of intersectoral collaborations for population health and to reduce health inequalities in high-income countries: A complexity-informed systematic review. Int J Health Policy Manag. 2022;11(12):2780-2792. doi:10.34172/ijhpm.2022.6550 PMC10105187 35219286

[39] Petticrew M Whitehead M Macintyre SJ Graham H Egan M . Evidence for public health policy on inequalities: 1: The reality according to policymakers. J Epidemiol Community Health. 2004;58(10):811-816. doi:10.1136/jech.2003.015289 PMC1763325 15365104

[40] Carey G Crammond B . Action on the social determinants of health: Views from inside the policy process. Soc Sci Med. 2015;128:134-141. doi:10.1016/j.socscimed.2015.01.024 25616195

[41] Carroll WK , ed. Regime of Obstruction: How Corporate Power Blocks Energy Democracy. Athabasca University Press; 2021.

[42] Wood B Lacy-Nichols J Sacks G . Taking on the corporate determinants of ill-health and health inequity: A scoping review of actions to address excessive corporate power to protect and promote the public’s health. Int J Health Policy Manag. 2023;12(1):1-17. doi:10.34172/ijhpm.2023.7304 PMC10590241 38618808

[43] Belliveau E . Big Oil’s lobbying playbook: A summary of the fossil fuel industry’s 2024 federal lobbying. Environmental Defence Canada; 2025, March.

[44] Romanello M di Napoli C Green C , et al. The 2023 report of the lancet countdown on health and climate change: The imperative for a health-centred response in a world facing irreversible harms. Lancet. 2023;402(10419):2346-2394. doi:https://doi.org/10.1016/S0140-6736(23)01859 10.1016/S0140-6736(23)01859-7 PMC7616810 37977174

[45] Legislative Assembly of Ontario. *Bill 47, Making Ontario Open for Business Act, 2018*. SO 2018, c 14. Published 2018. Accessed August 10, 2025. https://www.ontario.ca/laws/statute/s18014.

[46] Legislative Assembly of Ontario. *Bill 60, An Act to Amend and Enact Various Acts With Respect to the Health System*. Published May 2023. Accessed August 10, 2025. https://www.ola.org/sites/default/files/node-files/bill/document/pdf/2023/2023-05/b060ra_e.pdf.

[47] Government of Canada. Key Health Inequalities in Canada: A National Portrait. Public Health Agency of Canada; 2018.

[48] Borras J Goldring L Landolt P . Pandemic precarities: Immigration status, work, housing, and health among current and former non-status residents of Toronto. FCJ Refugee Centre and CEP Project; 2021.

[49] Borras AM . A critical political economy of health inequities. In: Primrose D Loeppky RD Chang R , eds. The Routledge Handbook of the Political Economy of Health and Health Care. Taylor & Francis; 2024:119–-1130.

[50] Borras AM . The political economy of racialized health inequities: A panoramic view. In: Bryant T , ed. Handbook on the Social Determinants of Health. Edward Elgar Publishing; 2025:190-203.

[51] Peters J , ed. Boom, Bust and Crisis: Labour, Corporate Power and Politics in Canada. Fernwood Publishing; 2012.

[52] Macdonald D . Born to Win. Canadian Centre for Policy Alternatives; 2018.

[53] Navarro V , ed. Neoliberalism, Globalization, and Inequalities. Routledge; 2007.

[54] Schrecker T Bambra C . How Politics Makes Us Sick: Neoliberal Epidemics. Springer; 2015.

[55] Bryant T Raphael D . The Politics of Health in the Canadian Welfare State. Canadian Scholars’ Press; 2020.

[56] Bryant T . Health Policy in Canada. 2nd ed. Canadian Scholars’ Press Inc; 2016.

[57] Borras AM . The challenge of exposing and ending health inequalities through social and policy change: Canadian experiences. Int J Soc Determinants Health Health Serv. 2023;53(2):130-145. doi:10.1177/27551938221148376 PMC11951455 40150794

[58] Das RJ . Capital, capitalism and health. Crit Sociol. 2023;49(3):395-414. doi:10.1177/08969205221083503

[59] Marx K Engels F . The Communist Manifesto. Moore S, trans. Simon & Schuster; 1964, Originally published 1848.

[60] Muntaner C Borrell C Ng E , et al. Politics, welfare regimes, and population health: Controversies and evidence. Sociol Health Illn. 2011;33(6):946-964. doi:10.1111/j.1467-9566.2011.01339.x 21899562

[61] Gunn V Kreshpaj B Matilla-Santander N , et al. Initiatives addressing precarious employment and its effects on workers’ health and well-being: A systematic review. Int J Environ Res Public Health. 2022;19(4):2232. doi:10.3390/ijerph19042232 PMC8872425 35206419

[62] Muller J Raphael D . Does unionization and working under collective agreements promote health? Health Promot Int. 2023;38(4):daab181. doi:10.1093/heapro/daab181 34897428

[63] O’Campo P Gunn V Perri M , et al. Working conditions, worker rights, and managerial domination during the COVID-19 pandemic: Assessing their toll on precariously employed workers and family well-being. Int J Soc Determinants Health Health Serv. 2024;54(3):233-246. doi:10.1177/27551938241253789 PMC11157980 38813773

[64] Kvart S Cuervo I Gunn V , et al. Labour and social protection gaps impacting the health and well-being of workers in non-standard employment: An international comparative study. PLoS One. 2025;20(3):e0320248. doi:10.1371/journal.pone.0320248 PMC11936240 40131914

[65] Evans B Fanelli C Panitch L Swartz D . From Consent to Coercion: The Continuing Assault on Labour. 4th ed. University of Toronto Press; 2023.

[66] Clarke J . Canadian unions and the state: Losing ground, moving on. *The Bullet*. Published April 9, 2023. Accessed May 10, 2025. https://socialistproject.ca/2023/04/canadian-unions-the-state-losing-ground-moving-on/.

[67] Azadian A Masciangelo MC Mendly-Zambo Z Taman A Raphael D . Corporate and business domination of food banks and food diversion schemes in Canada. Cap Class. 2023;47(2):291-317. doi:10.1177/03098168221092649

[68] Howlett M . Policy analytical capacity and evidence-based policy-making: Lessons from Canada. Can Public Adm. 2009;52(2):153-175. 10.1111/j.1754-7121.2009.00070_1.x

[69] Mackenbach JP . Has the English strategy to reduce health inequalities failed? Soc Sci Med. 2010;71(7):1249-1253. doi:10.1016/j.socscimed.2010.07.014 20736133

[70] Cairney P . The myth of ‘evidence-based policymaking’ in a decentred state. Public Policy Adm. 2020;37(1):46-66. doi:10.1177/0952076720905016

[71] Davey F McGowan V Birch J , et al. Levelling up health: A practical, evidence-based framework for reducing health inequalities. Public Health Pract. 2022;4:100322. doi:10.1016/j.puhip.2022.100322 PMC9494865 36164497

[72] Raphael D Sayani A . Assuming policy responsibility for health equity: Local public health action in Ontario, Canada. Health Promot Int. 2019;34(2):215-226. doi:10.1093/heapro/dax073 29092023

[73] Baum F Townsend B Fisher M , et al. Creating political will for action on health equity: Practical lessons for public health policy actors. Int J Health Policy Manag. 2022;11(7):947-960. doi:10.34172/ijhpm.2020.233 PMC9808180 33327689

[74] McIntyre L Shyleyko R Nicholson C , et al. Perceptions of the social determinants of health by two groups more and less affiliated with public health in Canada. BMC Res Notes. 2013;6:247. doi:10.1186/1756-0500-6-247 PMC3851988 23815886

[75] Friel S Townsend B Fisher M , et al. Power and the people’s health. Soc Sci Med. 2021;282:114173. doi:10.1016/j.socscimed.2021.114173 34192622

[76] McGrail K Morgan J Siddiqi A . Looking back and moving forward: Addressing health inequities after COVID-19. Lancet Reg Health Am. 2022;9:100232. doi:10.1016/j.lana.2022.100232 PMC8928332 35313508

[77] Reynolds MM Galea S . Advancing the study of power: Opportunities and priorities for understanding population health inequities. Am J Public Health. 2025;115(6):883-889. doi:https://doi.org/10.2105/AJPH.2025.308015 10.2105/AJPH.2025.308015 PMC12080447 40179346

[78] Card KG Hepburn KJ . Is neoliberalism killing us? A cross-sectional study of the impact of neoliberal beliefs on health and social wellbeing in the midst of the COVID-19 pandemic. Int J Soc Determinants Health Health Serv. 2022;53(3):363-373. doi:10.1177/00207314221134040 PMC9605858 36278290

[79] World Health Organization. World Report on Social Determinants of Health Equity. World Health Organization; 2025.

